# Individual Differences in Problem-Solving Behavior in Reasoning Tests: The Role of Item Difficulty and Item Position

**DOI:** 10.3390/jintelligence14090206

**Published:** 2026-09-01

**Authors:** Helene M. von Gugelberg

**Affiliations:** Institute of Education, University of Zurich, 8001 Zurich, Switzerland; helene.vongugelberg@ife.uzh.ch

**Keywords:** individual differences, reasoning, working memory, eye-tracking, item-position effect

## Abstract

Matrix reasoning performance is often treated as a static indicator of cognitive ability, although it reflects a dynamic process that unfolds across items. The present study examined how reasoning ability and visuospatial working memory (vsWM) interact with item difficulty and item position to shape problem-solving behavior. Eye movements were recorded from 300 participants during a figural matrices test. Three indices of problem-solving behavior were analyzed using Bayesian multilevel models: toggle rate (ToR), proportional time on matrix (PoM), and proportional time to first fixation on response alternatives (PoFA). Results showed that higher reasoning was consistently associated with behavior indicative of constructive matching, reflected in lower ToR, higher PoM, and higher PoFA. Further, individuals with higher reasoning ability showed stronger adaptations in problem-solving behavior in response to changing task demands. Evidence regarding vsWM was more nuanced, revealing selective interactions with reasoning ability and test characteristics rather than a consistent main effect across measures. Item position revealed substantial between-person variability, which exceeded the variability associated with item difficulty. This suggests that individuals differ more strongly in how their behavior evolves across the test than in their responses to increasing difficulty. Overall, the findings highlight that problem-solving behavior in reasoning tests reflects a dynamic interplay between individual characteristics and test characteristics, emphasizing the importance of modeling both simultaneously when investigating reasoning performance.

## 1. Introduction

**Figure 1:**
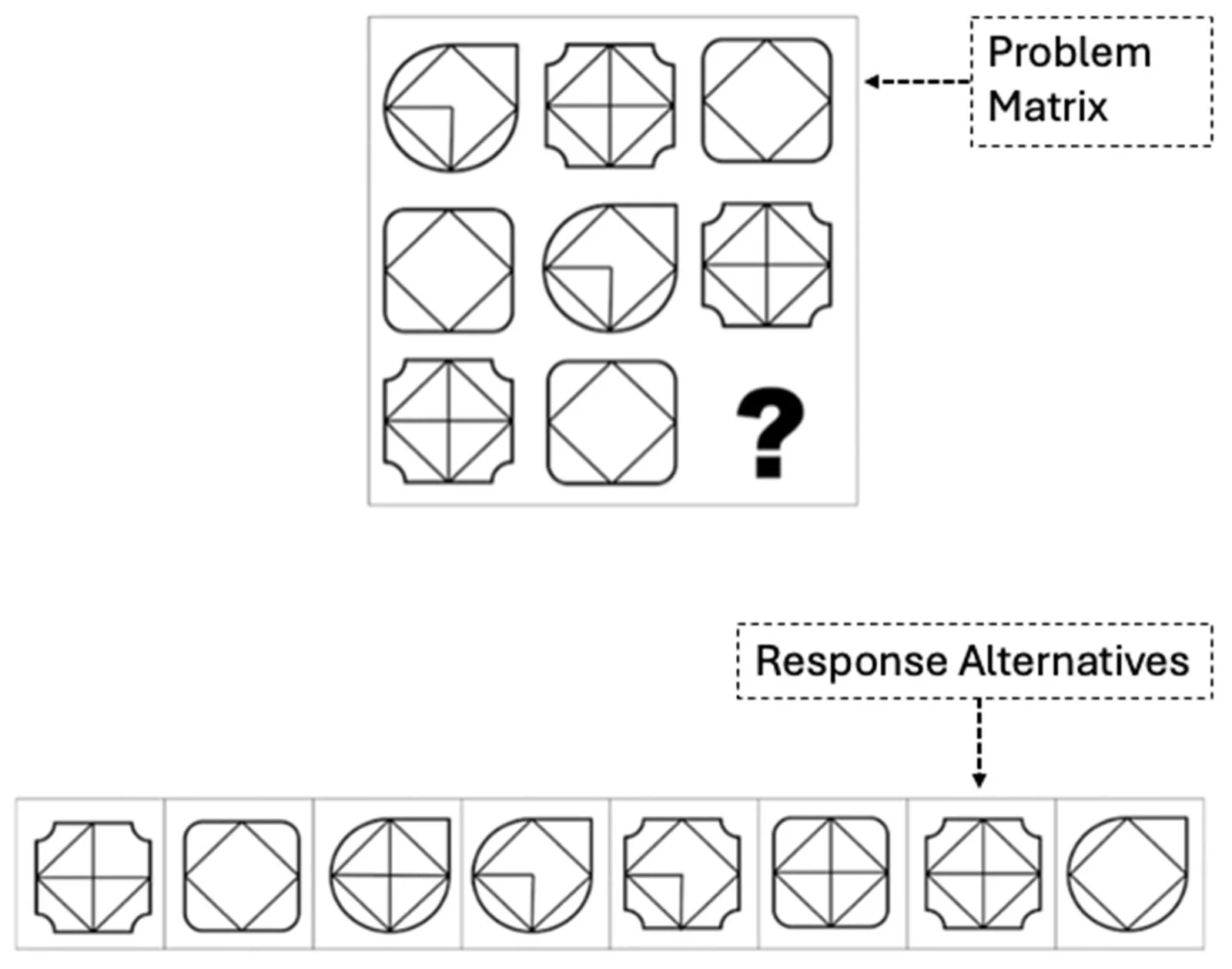
Reasoning ability test item from the Figural Matrices Test (Kyllonen et al., 2019). Dashed arrows and text boxes were added for explanatory purposes only. A detailed test description is given in Section 2.

Matrix reasoning tests are widely recognized as good predictors for future success in various domains, including educational achievement (Deary et al., 2007; Roth et al., 2015) and professional performance (Sackett et al., 2024). A typical matrix reasoning test item (see Figure 1) consists of a problem matrix with one entry missing, and the individual is tasked with identifying the missing piece from a set of provided response alternatives.

Despite their prevalence, individual differences in problem-solving behavior during test-taking are still not fully understood and have fascinated researchers for many years. Snow’s (1978, 1980) work provided an early basis for examining how individuals apply strategies during reasoning tasks. In these studies, participants completed eight cognitive ability tests including the Raven’s progressive matrices (RPM; Raven & Raven, 2003), while their eye movements were recorded. After completing the RPM, participants described the strategies they used both for specific items and their general approach to test-taking. Snow then analyzed performance data, eye-movement patterns, and introspective reports to identify problem-solving behavior.

Snow (1978) observed one strategy that was most consistently associated with higher reasoning ability test scores and later labeled it constructive matching (Snow, 1980). With this strategy, individuals reported mentally constructing a potential solution based on the problem matrix and then quickly scanning the response alternatives to identify the best match. Correspondingly, their eye movement showed intensive inspection of the matrix and relatively little attention to the response alternatives. In contrast, lower ability individuals tended to exhibit frequent shifts between the matrix and the response alternatives, a pattern labeled response elimination.

Snow also observed substantial variability in how consistently individuals applied these strategies. Some participants relied on a single approach across all items, whereas others adapted their strategies when their initial approach was ineffective. Notably, Snow did not find a clear pattern indicating whether high- or low-ability individuals were more likely to switch strategies during the task.

Roughly 50 years later, scientific advances have made eye-tracking measures and statistical analyses more sophisticated. The distinction between constructive matching (CM) and response elimination (RE) prevailed but has also been expanded as additional strategies and hybrid approaches have been identified.

For example, Jarosz et al. (2019) discovered a third strategy they named isolate-and-eliminate. Their data revealed that this problem-solving behavior was somewhat of a hybrid approach between CM and RE, where individuals isolate a specific rule in the problem matrix and then proceed to eliminate non-viable response alternatives regarding this rule, and repeat this procedure until only one response remained. With this strategy individuals construct partial responses, while RE does not include any mental construction of responses.

Such strategic nuances suggest that problem-solving behavior is likely not a binary choice between CM and RE. These two strategies might rather characterize two specific extremes, whereas an individuals’ problem-solving behavior likely lies somewhere in between.

The presence of a third strategy was later supported by the findings of Li et al. (2022). They ran a latent profile analysis on answers from a strategy questionnaire, discovering that individual differences in response patterns can be better described by three, instead of only two groups. In addition to the two groups giving answers corresponding either to CM or RE, the third group provided answers indicating problem-solving behavior typical for both strategies. These three groups also showed different patterns in their eye-tracking metrics.

Three different eye-tracking metrics are often reported to assess problem-solving behavior in matrix reasoning tasks. Toggle Rate (ToR) accounts for all switches an individual makes between the problem matrix and response alternatives divided by item latency. Hence, it is the absolute number of switches/toggles an individual makes while solving an item, taking into account how long it takes the individual to solve said item. Fewer switches are said to indicate CM, as individuals consult response alternatives less during problem-solving (e.g., Laurence et al., 2023).

Proportional time on matrix (PoM) is the metric indicating how much time an individual spent investigating the problem matrix proportional to item latency. Higher values would indicate CM, as proportionally more time is spent investigating the problem matrix compared to any other aspects of the item (e.g., Raden & Jarosz, 2022).

Further, proportional time to first fixation on response alternatives (PoFA) captures the first moment an individual consults the response alternatives divided by item latency. Here, larger values would also be in line with CM, as an individual spent more time investigating the problem matrix before looking at possible solutions within the response alternatives (e.g., Vigneau et al., 2006).

These associations between CM and the three eye-tracking metrics were also evident in Li et al. (2022). Their CM group showed significantly higher PoM and PoFA, and lower ToR compared to the two other groups. In addition to differences in self-reported strategy use and eye-tracking metrics, the groups also varied significantly in their RPM scores, with the CM group outperforming the other two groups. Across numerous studies (see Table 1), problem-solving behavior more akin to CM has been related to higher reasoning ability. This suggests that reasoning ability might be one factor as to why individuals engage in different problem-solving behaviors.

Given the robust association between reasoning ability and CM across studies, it is unsurprising that researchers have investigated whether the correlation between reasoning and CM could account for the well-established link between reasoning ability and working memory (e.g., Kane et al., 2005). Evidence remains mixed. The relation between working memory capacity (WMC) and reasoning has been found to be moderated (Li et al., 2022) or mediated (Gonthier & Thomassin, 2015; Jarosz et al., 2019) by strategy use, while another study found neither (Jastrzębski et al., 2018).

Nevertheless, the findings remain theoretically consistent with WMC exhibiting a negative correlation with ToR (e.g., Raden & Jarosz, 2022; Laurence et al., 2023), indicating a stronger degree of CM when WMC is high. WMC entails the ability to build, hold, and manipulate temporary representations (Oberauer, 2020, p. 118), and CM is just that. To mentally construct responses, individuals must build mental representations, hold them and relevant rules of an item in mind, and manipulate the mental representation until a satisfactory solution is obtained. Hence, it is not surprising that individuals with higher scores in a visuospatial working memory (vsWM) task also exhibited eye movements in line with CM (Laurence et al., 2023).

With increasing item difficulty, more rules and item features must be maintained simultaneously (Carpenter et al., 1990), making CM increasingly resource demanding. Early findings of Bethell-Fox et al. (1984) showed that with increasing item difficulty, individuals showed a decrease in CM. This shift was assumed to be due to limited WMC. Whenever an individual reached their WMC limit, they would shift to less demanding RE. A shift from CM to more RE with increasing item difficulty was also observed by Gonthier and Roulin (2020). However, somewhat the opposite was found by Niebaum and Munakata (2023).

This is where the conundrum surrounding problem-solving strategies in reasoning tests continues, as studies incorporating item difficulty have reported conflicting findings. For example, Niebaum and Munakata (2023) found that time to first fixation on response alternatives increased with item difficulty, suggesting greater CM in adults and in 6- and 9-year-olds. Similarly, ToR decreased with increasing item difficulty, although an increase in ToR among 9-year-olds complicated the interpretation. Gonthier et al. (2024) likewise reported greater CM for more difficult items across PoFA, PoM, and ToR in participants aged 6 to 18 years.

In adults, von Gugelberg and Troche (2025) also observed a shift toward greater CM with increasing item difficulty based on ToR and PoM. However, PoFA decreased, indicating less CM. In contrast, Vigneau et al. (2006) found no association between item difficulty and problem-solving behavior after accounting for item latency (i.e., time spent working on an item). These inconsistencies may reflect differences in sample characteristics, test settings (e.g., item type, time limits), and individual differences in problem-solving behavior.

The assumption that individual differences might at least partially explain diverging results regarding problem-solving behavior is supported by evidence indicating that a shift in strategy is modulated by individual differences in reasoning ability. Results from Liu et al. (2023) showed that participants with high reasoning ability increased their use of CM as item difficulty rose, and participants with low reasoning decreased its use. Hence, their results portray opposite direction of effects regarding item difficulty depending on reasoning ability.

The role of reasoning ability is also evident in other aspects of problem-solving behavior. For example, participants with high reasoning ability seem to exhibit stronger modulation in test-taking strategy (Diu et al., 2025) and greater changes in item latency with increasing item difficulty (Cheyette & Piantadosi, 2024; von Gugelberg & Troche, 2025). Similarly, when comparing different age groups, as reasoning ability increases with age, studies showed that older children modulate their item latencies to a stronger degree from easy to difficult items (Gonthier et al., 2024; Perret & Dauvier, 2018).

It seems that item difficulty is a driving factor concerning problem-solving behavior and that an individual’s reasoning ability and/or WMC contributes to how individuals react to item difficulty. With many reasoning tests relying on progressive item difficulty, it is a challenging endeavor to distinguish effects of item difficulty and item position. Nevertheless, several studies highlight how effects of item position can explain variance in reasoning tests beyond reasoning ability (Schweizer, 2006) and how the item-position effect is distinct from item difficulty (Zeller et al., 2017). The item-position effect can also serve as a predictor of school achievement above and beyond reasoning ability (Ren et al., 2014), reinforcing the assumption that the item-position effect is not a methodological artefact but a psychologically meaningful entity.

Several accounts have been proposed for the item-position effect. One interpretation emphasizes the process of meaning-making during test taking (Knowles et al., 1996). When solving a series of similar items, the test taker might increasingly be able to understand what the goal of the measurement entails. A better understanding might allow for the test taker to be better able to distinguish different features, or perceive nuances overlooked at the beginning.

Specifically for reasoning tests, the learning hypothesis was proposed for the item-position effect (Ren et al., 2014; von Gugelberg et al., 2025). This hypothesis goes beyond meaning-making and delves into the ability to learn during test taking. Here, the assumption holds that individual differences in the ability to learn rules during test-taking are one additional major source of variance in the data. Hence, if a certain item was presented early during the test, individuals might show different problem-solving behavior compared to when the same item would have been displayed toward the end of the test, as individuals had a chance to learn the underlying rules of previous test items. Further, this change in behavior varies between individuals, as they exhibit different degrees of learning during test taking. Therefore, to fully address problem-solving behavior in reasoning tests, item position must be taken into account.

Taken together, existing research suggests that problem-solving behavior in matrix reasoning tasks reflects a dynamic interplay between individual differences (e.g., reasoning ability and vsWM) and task characteristics such as item difficulty and item position. At the same time, findings across studies are not always consistent, which likely reflects differences in test materials, sample characteristics, and methodological approaches. As a result, it remains difficult to draw conclusive inferences regarding how these factors jointly shape strategy use during test taking. The present study addresses this issue by adopting a Bayesian multilevel modeling approach. Multilevel modeling allows for the simultaneous modeling of both within-person changes across items and between-person variability in problem-solving behavior, thereby explicitly accounting for individual differences in test taking. Within this framework, the goal is to examine how reasoning ability and vsWM interact with item difficulty and item position to influence problem-solving behavior. In doing so, the study aims to contribute to a more integrated understanding of how cognitive ability and task structure jointly shape strategy use in reasoning tests.

## 2. Materials and Methods

### 2.1. Participants

Participants were recruited via the university, local institutions, online channels, and directly by project contributors. Psychology undergraduates received course credit, whereas participants without a university entrance qualification were compensated with 20 CHF. The study was conducted in two separate sessions at least one day apart. A total, 319 individuals took part. For 312 participants eye-tracking calibration was successful^1^, nevertheless, thereof only 301 participants had reasonable fixations on stimuli^2^. Further, one additional participant had to be excluded as no data were recorded due to a computer issue during the vsWM task.

Hence, the final sample used for the analysis consists of 300 participants. This sample had a mean age of 26.26 years (*SD* = 10.81). Thereof, 183 identified as female, 112 as male, and two as “other.” Three participants chose not to report age, gender, or education. Regarding education, 209 participants reported a university entrance qualification as their highest degree, 59 held a university degree, and 29 reported neither. All participants had normal or corrected-to-normal vision and provided written informed consent. The study was approved by the local ethics committee of the Faculty of Human Sciences at the University of Bern (No. 2020-07-00001).

### 2.2. Figural Matrices

Form 1 of the Figural Matrices (FM) developed as part of the Mitres battery (Kyllonen et al., 2019) was administered. For the FM, item difficulty is not progressive and varies throughout the test. Each FM item consisted of a black-and-white 3 × 3 matrix containing eight geometric figures, with the lower right cell missing. Eight response options were displayed below the matrix (see Figure 1). The problem matrix measured 10.8 cm in width and 11.6 cm in height, while the area containing the response alternatives measured 26.3 cm by 3.5 cm. Responses were provided by selecting one of the alternatives using a wired computer mouse. The following instructions were consistent with the test manual, and participants completed two practice items prior to the test. As specified in the manual, a time limit of 30 min was set and an additional time limit of 2 min for each item was implemented. Responses were scored as correct or incorrect.

### 2.3. Corsi Block-Tapping Task

The Corsi block-tapping task (CBT) was used to assess visuospatial working memory (vsWM). Participants were presented with nine white squares, spatially distributed on a gray screen. On each trial, a set of squares was highlighted sequentially. This sequence then had to be reproduced in the same order by clicking on the respective squares. Sequences increased in length from three to nine items, with three trials per sequence, resulting in a total of 21 trials. The positions of highlighted squares were pseudorandomized. Responses were scored as correct or incorrect, and the total number of correct trials served as the performance measure.

### 2.4. Eye-Tracking Metrics

Participants were positioned 85 cm from the screen, with viewing distance controlled by a chin-forehead rest. Eye movements were recorded using an EyeLink 1000 Plus eye-tracking system (SR Research, 2016) with an infrared video-based camera sampling at 500 Hz. Analyses were conducted on monocular data, with the eye showing lower measurement error automatically selected for analysis. A minimum fixation duration threshold of 100 ms was applied (e.g., Martarelli & Mast, 2013). Areas of interest (AOIs) were defined for the problem matrix and the response alternatives. Additional padding was applied to the AOI surrounding the response alternatives (Bojko, 2013), whereas no padding was added to the matrix AOI because sufficient empty space was already present around the matrix elements (see Figure 1).

Eye-tracking data for each participant and item were visually inspected for drift using EyeLink Data Viewer software (SR Research EyeLink: Ottawa, ON, Canada). Drift can occur if participants move their head after calibration, resulting in a systematic displacement of fixations toward blank areas on the screen. When such drift was detected, fixations were adjusted uniformly to relocate them to more plausible areas.

Proportional time on the problem matrix (PoM) was computed by adding up the durations of all fixations within the matrix AOI and dividing this value by item latency. The first fixation on response alternatives was directly extracted from the EyeLink Data Viewer. Proportional time to first fixation on response alternatives (PoFA) was calculated by dividing the extracted value by item latency.

Toggle rate (ToR) was calculated based on fixation reports generated for each participant. A toggle was defined as a transition between the problem matrix AOI and the response alternatives AOI, or vice versa. Fixations occurring outside the predefined AOIs were recoded as belonging to the AOI of the immediately preceding fixation. This procedure ensured that scan paths involving brief fixations on blank screen areas (e.g., during pauses in processing) were still counted as toggles. For each item, the total number of toggles was divided by item latency to yield the ToR.

### 2.5. Procedure

Reported data were part of a larger study with each participant completing two separate sessions. Data during both sessions were collected using PsychoPy v2020.1.2 (Peirce et al., 2019) on a desktop computer equipped with an 18-inch Dell monitor (resolution: 1280 × 1024 pixels), measuring 34 cm in width and 27 cm in height. During the first session, each participant completed various experimental tasks including the CBT task while the experimenter was present, in the event of technical issues.

The first session concluded after participants completed a demographic questionnaire and were reminded about the time and date for the second session. The second session took place at least 1 day but at a maximum of 8 days after the first session. The second session began with a brief explanation of the eye-tracking and other biometric sensors. Participants were instructed to find a comfortable position in the chair, to rest their chin on the chinrest, and to ensure they could operate the computer mouse well. As soon as participants felt comfortable and were properly positioned in the chin-forehead rest, the experiment started with the calibration of the eye-tracking. After successful calibration participants completed a reasoning task not analyzed in the current manuscript. After 30 min or when the task was completed, participants were informed that they were halfway through the second session. All participants were encouraged to take a short break, stand up for a moment, stretch their legs and drink some water. Thereafter, all biometric sensors were reattached, and the calibration for the eye-tracking was repeated. After successful calibration, participants were presented with the FM.

For the FM, the remaining time for test completion was displayed on the screen. Participants were also instructed that the short interval between items (2 s) did not contribute to the time limit and that they should simply look at the fixation cross displayed during that time. After completing the FM, the second session ended, and participants were debriefed and thanked for their participation.

Data were collected in two separate batches due to administrative reasons. The first batch contained eye-tracking data of 217 participants. After a short break of a couple of months, data collection resumed, and data of a further 95 participants were collected.

### 2.6. Statistical Analysis

The eye-tracking data were processed and corrected using EyeLink’s Data Viewer software (SR Research, 2016). Subsequent data analysis was performed using R version 4.0.2 (R Core Team, 2020). The *tidyverse* package (v1.3.1; Wickham et al., 2019) was utilized for data processing, preparation, and descriptive statistics. Pearson product-moment correlations were calculated between eye-tracking strategy indices and ability scores, and these were compared with previous study results. Multilevel models were estimated using the *brms* package (v2.23.0; Bürkner, 2017).

#### Bayesian Multilevel Models

Data were analyzed using a Bayesian multilevel linear regression model. The predictors vsWM, reasoning ability, and item difficulty^3^ were scaled prior to analysis (mean = 0, *SD* = 1), such that fixed effects represent associations at mean levels of the other predictors. Item position was grand-mean centered and divided by 10. Hence, effects represent associations for items positioned in the middle of the test. Main effects included visuospatial working memory (vsWM), reasoning ability, item difficulty, and item position with all possible interactions up to the four-way level.

At the between-participant level, the model included random intercepts to capture individual differences in the overall outcome. To allow the effects of test characteristics to vary across individuals, random slopes for item difficulty and item position were estimated. Correlations among random intercepts and random slopes were also estimated, allowing for a fully correlated random-effects structure.

Models were estimated using Hamiltonian Monte Carlo as implemented in *brms* (Bürkner, 2017), with four chains and 8000 iterations per chain (4000 warm-up), yielding 16,000 post–warm-up samples. Convergence was assessed using $\hat{R}$, effective sample sizes, and visual inspection of trace plots (see Supplementary Materials on OSF for full model specifications, including trace and density plots and posterior predictive checks).

Fixed effects were assigned flat priors, participant-level random effects were assigned a weakly informative Student-*t* prior, and group-level standard deviation parameters were assigned half–Student-*t* priors. Correlations among group-level effects were assigned a uniform prior over correlation matrices, unless specified otherwise. Posterior estimates are reported with 95% credible intervals. Effects are considered credible when intervals exclude zero.

Models were initially estimated assuming a Gaussian distribution with an identity link function for the outcome variable. Posterior predictive checks for the ToR model indicated potential model misfit, particularly due to skewness and heteroskedasticity. Consistent with the observed density, models with hurdle gamma and hurdle lognormal distributions (hurdled to account for meaningful values of zero) were also estimated and compared using leave-one-out cross-validation. Model comparison indicated that the hurdle gamma model with a log link function provided the best fit and was therefore retained for ToR.

For PoM and PoFA, the Gaussian distribution with an identity link function provided an adequate fit, and no adjustments were made. Detailed model estimates, convergence diagnostics, and posterior predictive checks for both the initial and final models are provided in the Supplementary Materials on OSF.

## 3. Results

**Figure 2:**
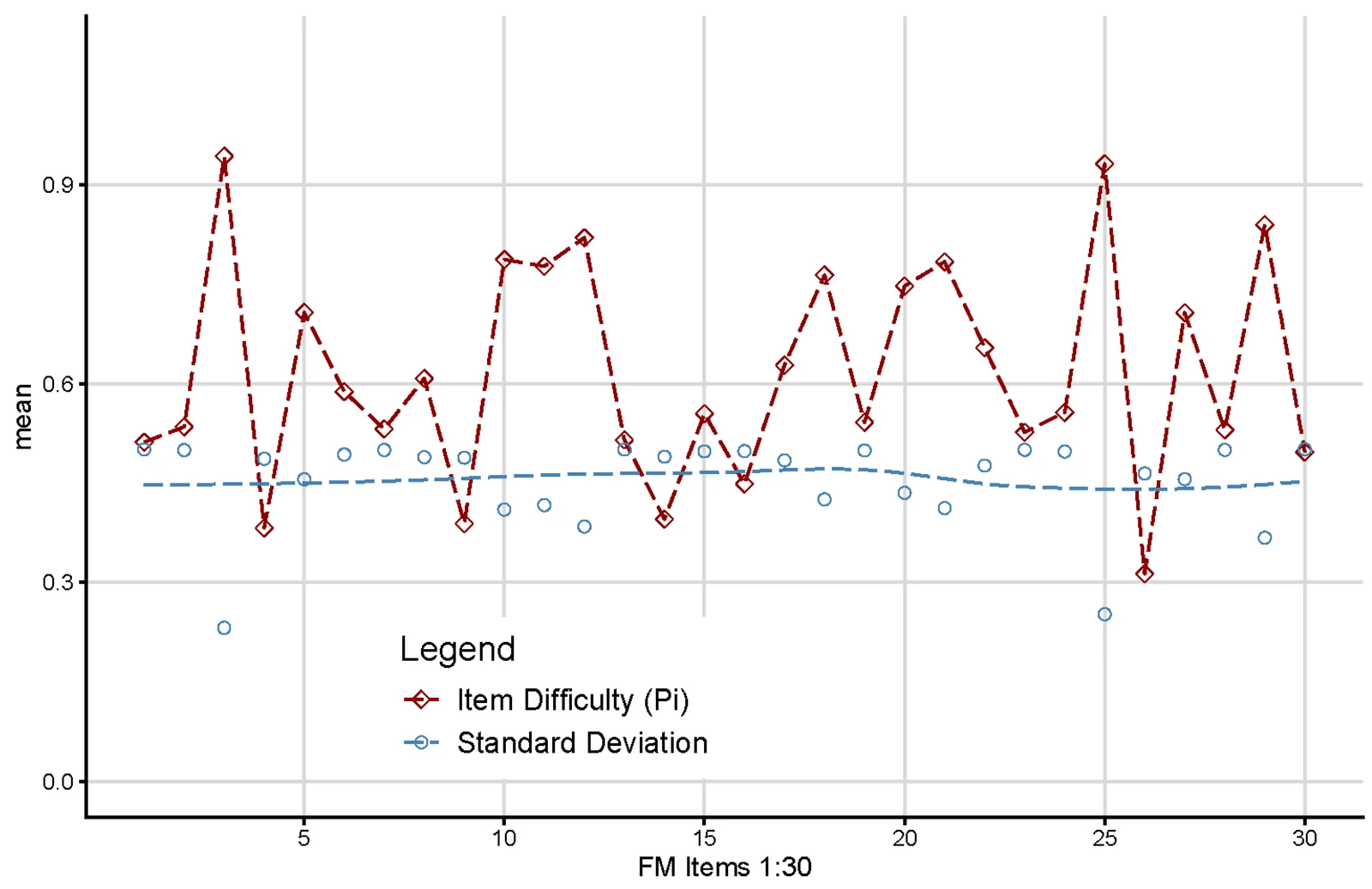
Item difficulties (P_i_) depicted as squares connected by a dashed line and circles indicating standard deviation connected by a dashed trend line for all items of the figural matrices test.

**Figure 3:**
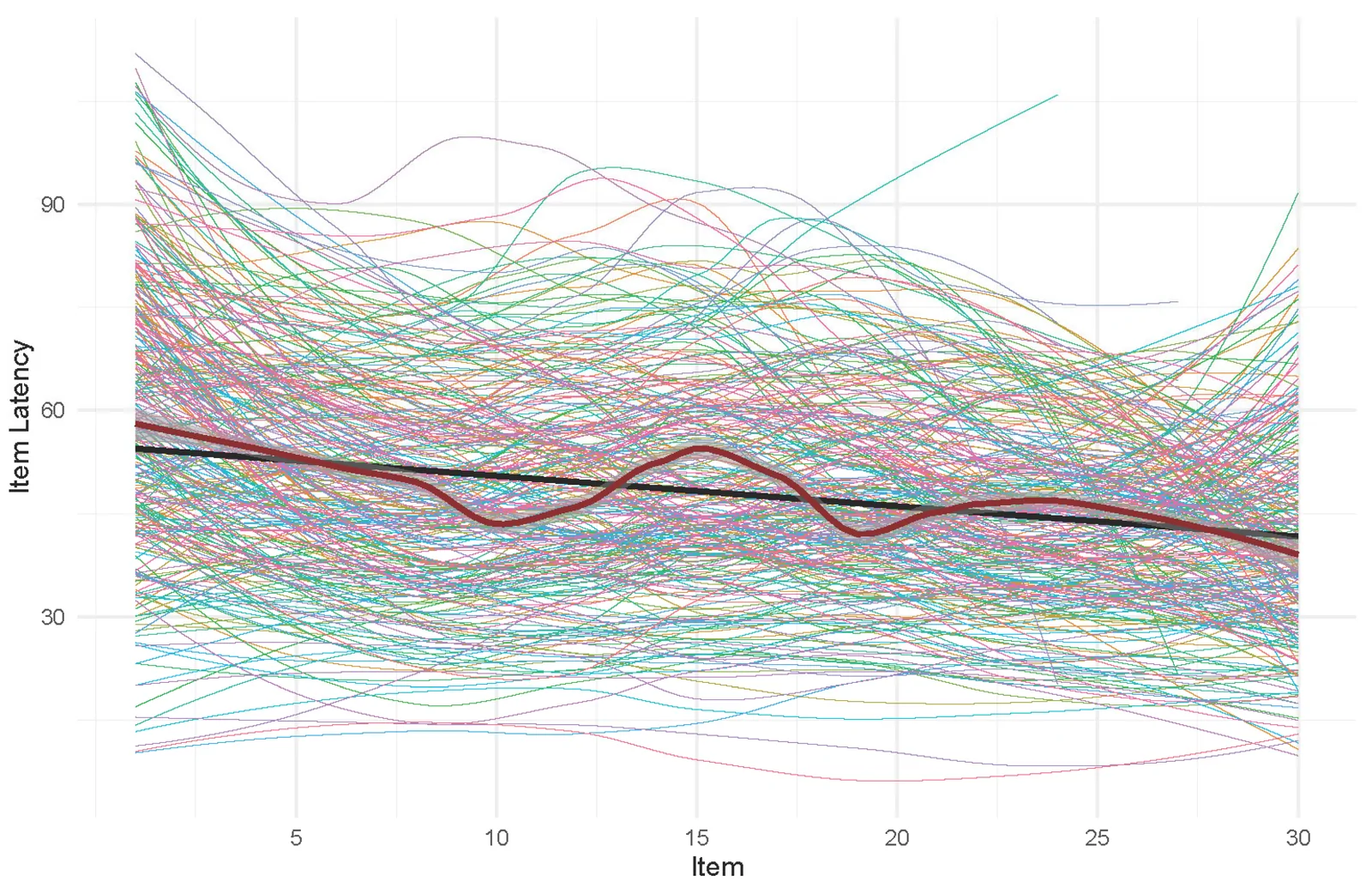
Given are item latencies (time spent on an item) for each individual, a linear (black) and local polynomial (red) regression line across all items of the figural matrices test.

Data were collected as part of a larger project. From the 319 participants in the final sample, 19 had incomplete data and were removed from all further analyses. Hence, reported results include 300 participants. Measuring vsWM, the mean score on the CBT was 11.96 (*SD* = 2.87). As depicted in Figure 2, item difficulty in the FM varied as the test progressed. In the current sample, participants took an average of 22.93 min (*SD* = 5.99) to solve the FM. Item latency (i.e., time spent working on an item) showed a slight downward trend when inspecting the linear regression line plotted in Figure 3. This decrease is distinctly different from what other studies reported (e.g., Gonthier & Roulin, 2020; Vigneau et al., 2006; von Gugelberg & Troche, 2025), with one driving factor most likely being that item difficulty did not increase from item to item in the current test as it frequently did in other studies as they implemented a version of the APM.

Correlations of the different eye-tracking metrics with reasoning ability are similar to other studies (see Table 1 and Table 2). The correlation of vsWM with reasoning ability was similar to what Raden and Jarosz (2022) reported, where they used a variation of operation span tasks, and to the example Laurence et al. (2023) reported, where they also implemented the CBT. Further, no significant correlation between time to first fixation on response alternatives and vsWM occurred. Number of toggles showed a small negative correlation in the current study, similarly to Laurence et al. (2023), where it was a medium negative correlation. Not in line with previous findings is the negative correlation between ToR and vsWM in the current study. This correlation was not significant in Laurence et al. (2023) and in Raden and Jarosz (2022) only reached significance in one of the reported conditions. Another discrepancy is that Laurence et al. (2023) found a negative correlation between vsWM and item latency, while this correlation was not significant in the current study.

### 3.1. Toggle Rate

To ease interpretability, the outcome variable ToR was rescaled by a factor of 100 prior to analysis. This linear transformation does not affect parameter estimates apart from shifting the intercept. Further, the model assuming a Gaussian distribution did not adequately capture the distribution of the outcome variable. A hurdle gamma distribution improved model fit and was therefore retained (see posterior predictive checks for each model in the Supplementary Materials provided on OSF).

**Figure 4:**
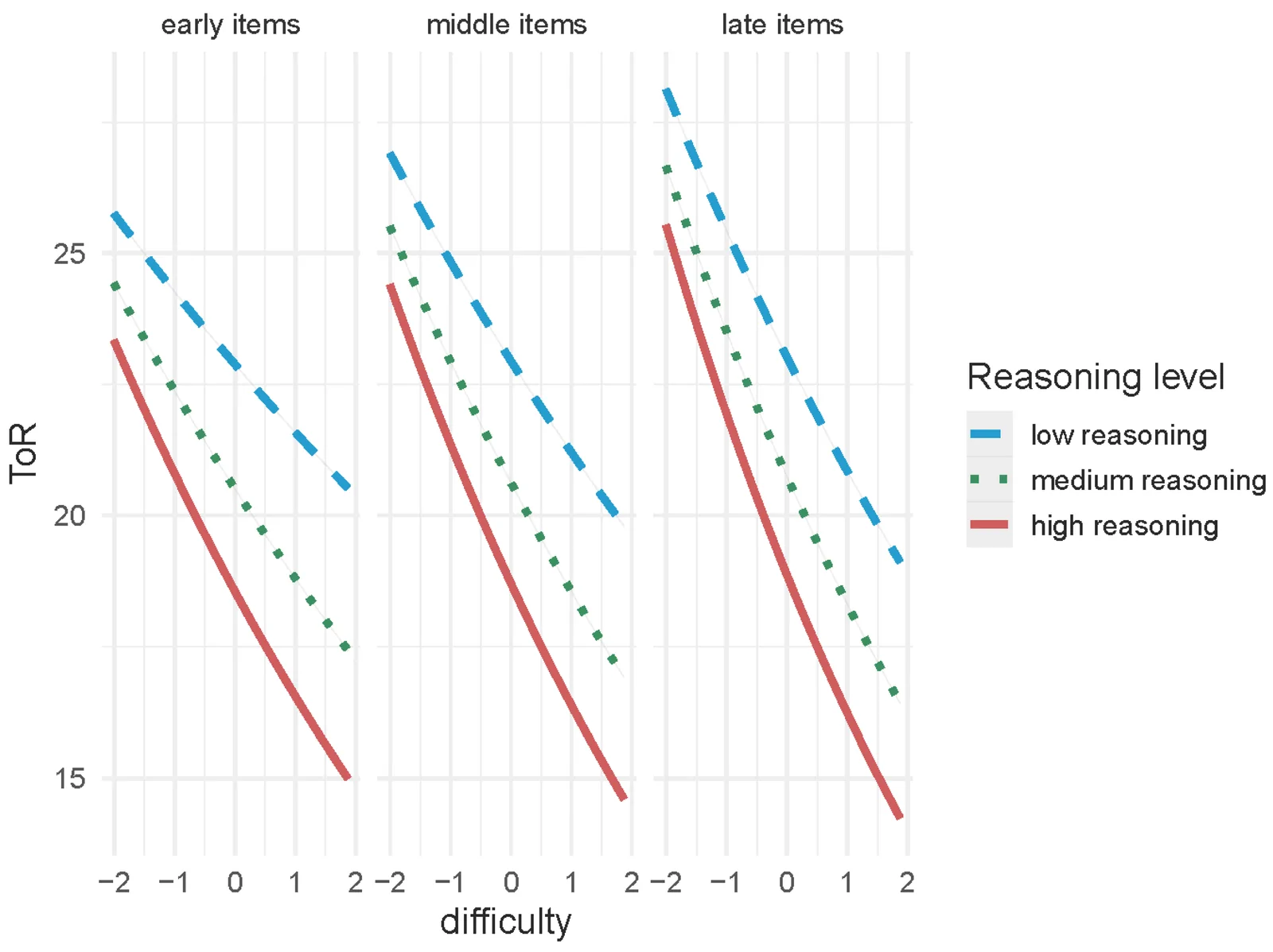
Toggle Rate across item position, varying levels of item difficulty and reasoning ability.

The model indicated no credible main effect for vsWM and item position (see Table 3). Higher reasoning ability and higher item difficulty were both associated with lower ToR. This is illustrated in Figure 4, where the interaction between the two is also visible. The change in ToR for difficult items was larger for participants with higher reasoning ability. Further, Figure 4 nicely illustrates the interaction between item difficulty and item position. The difference in ToR between easy and difficult items increased as the test progressed. Further, the model revealed an interaction between reasoning ability and vsWM (not illustrated in Figure 4), indicating that with higher vsWM, the effect of reasoning ability on ToR shrank. This means that the main negative effect of reasoning ability was moderated by vsWM, as with each unit of increase in vsWM the decrease in ToR due to reasoning was reduced. For all other interactions, the model provided no credible evidence.

**Figure 5:**
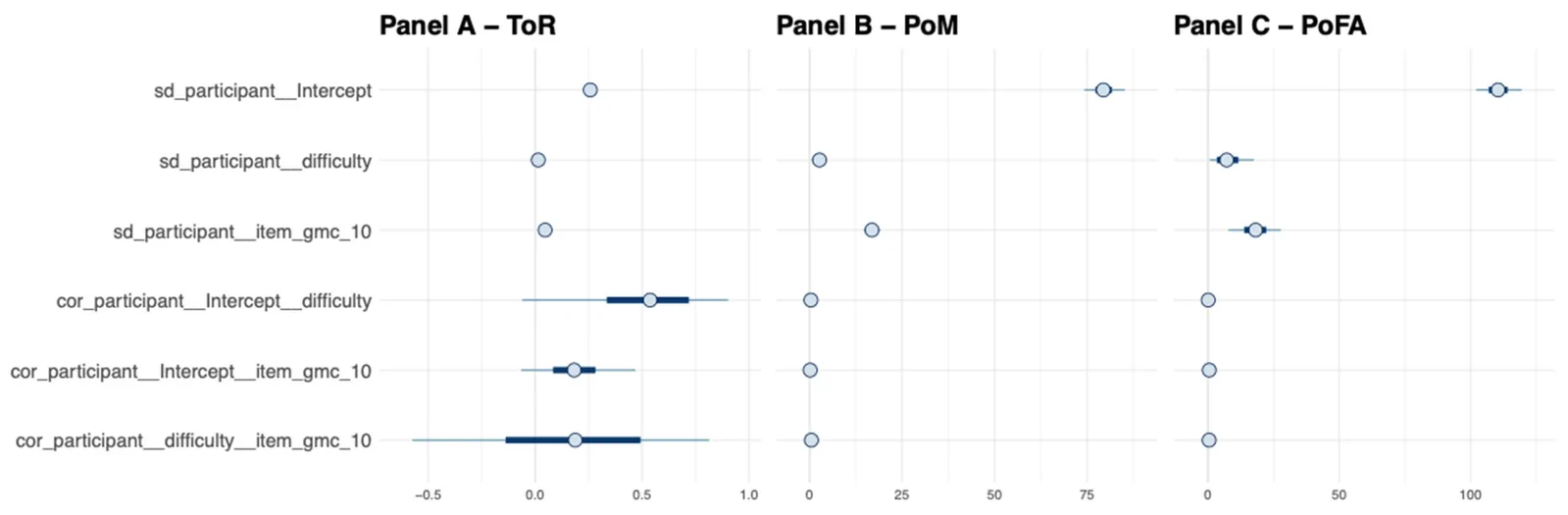
Posterior interval estimates of random effects for each Bayesian model. Each panel displays posterior means (circles) and 95% credible intervals (lines) for the random-effects parameters. Credible intervals excluding zero indicate evidence for non-zero effects. Panel A corresponds to toggle rate (ToR), Panel B to proportional time on matrix (PoM), and Panel C to proportional time to first fixation on response alternatives (PoFA). Item position was grand-mean centered and divided by 10 as indicated by the label item_gmc_10. All parameters demonstrated satisfactory convergence ($\hat{R}$ ≤ 1.01).

Random effects (displayed in Figure 5) show that the slopes between individuals varied depending on item position and item difficulty. The effect of item difficulty, while still meaningful, is somewhat smaller. Hence, individuals differ in how they adapt their problem-solving behavior to changing item difficulty and item position, with the latter being more pronounced.

### 3.2. Proportional Time on Matrix

**Figure 6:**
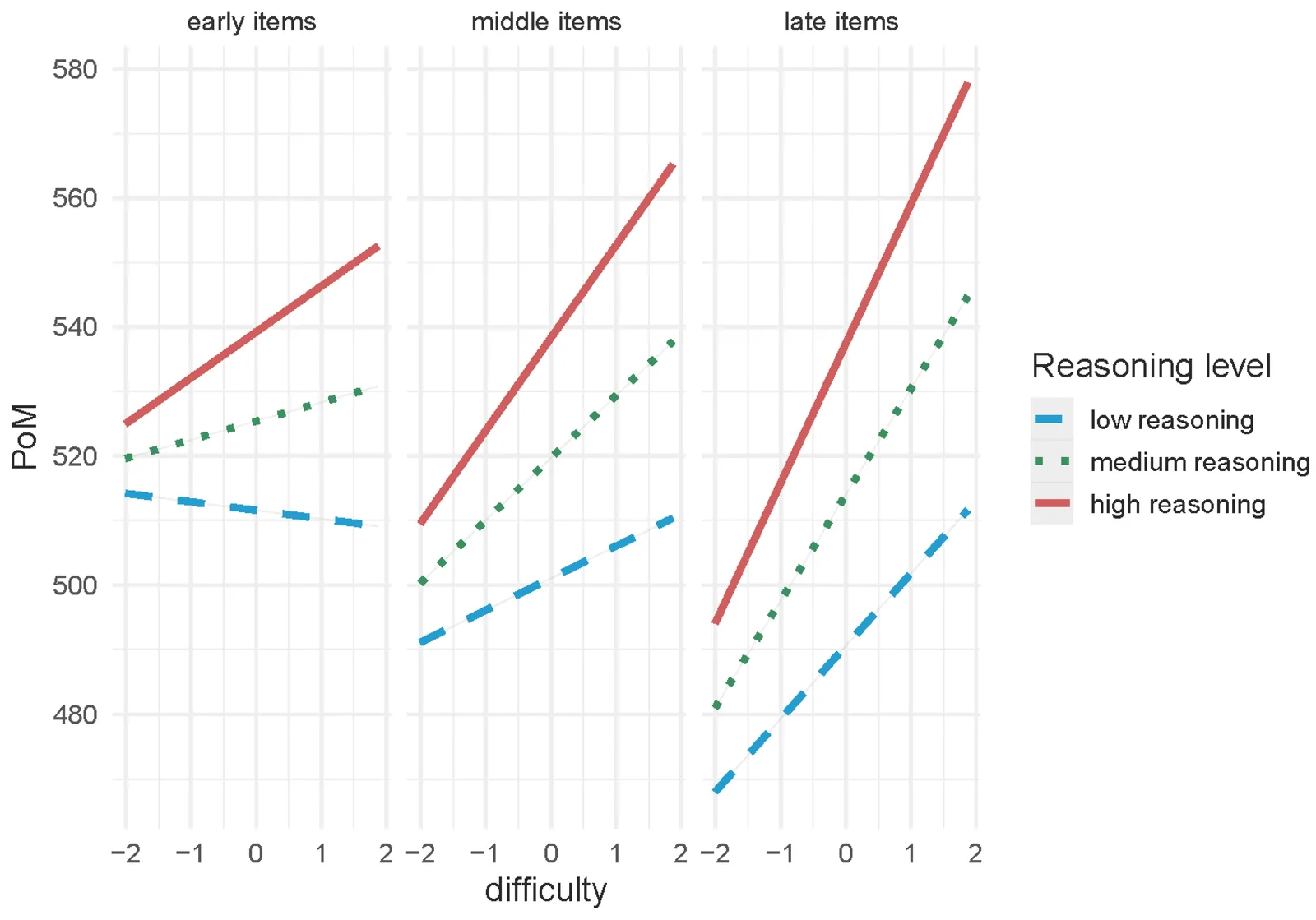
Proportional time on matrix across item position, varying levels of item difficulty and reasoning ability.

Regarding PoM, there was a credible main effect of reasoning, item difficulty, and item position, but not vsWM (see Table 4). With higher reasoning ability PoM increased, as was the case for item difficulty. For item position, PoM decreased, meaning that as the test progressed, participants spent proportionally less time investigating the matrix. The posterior estimates further provided credible evidence for an interaction between reasoning ability and item difficulty. Hence, as for ToR, the change in problem-solving behavior regarding item difficulty was larger for participants with higher reasoning ability. This effect on PoM is illustrated in Figure 6. The figure also highlights that the effect of reasoning and item difficulty changed as the test progressed. This was further supported by credible evidence for both the interactions of reasoning ability and item position and difficulty and item position.

**Figure 7:**
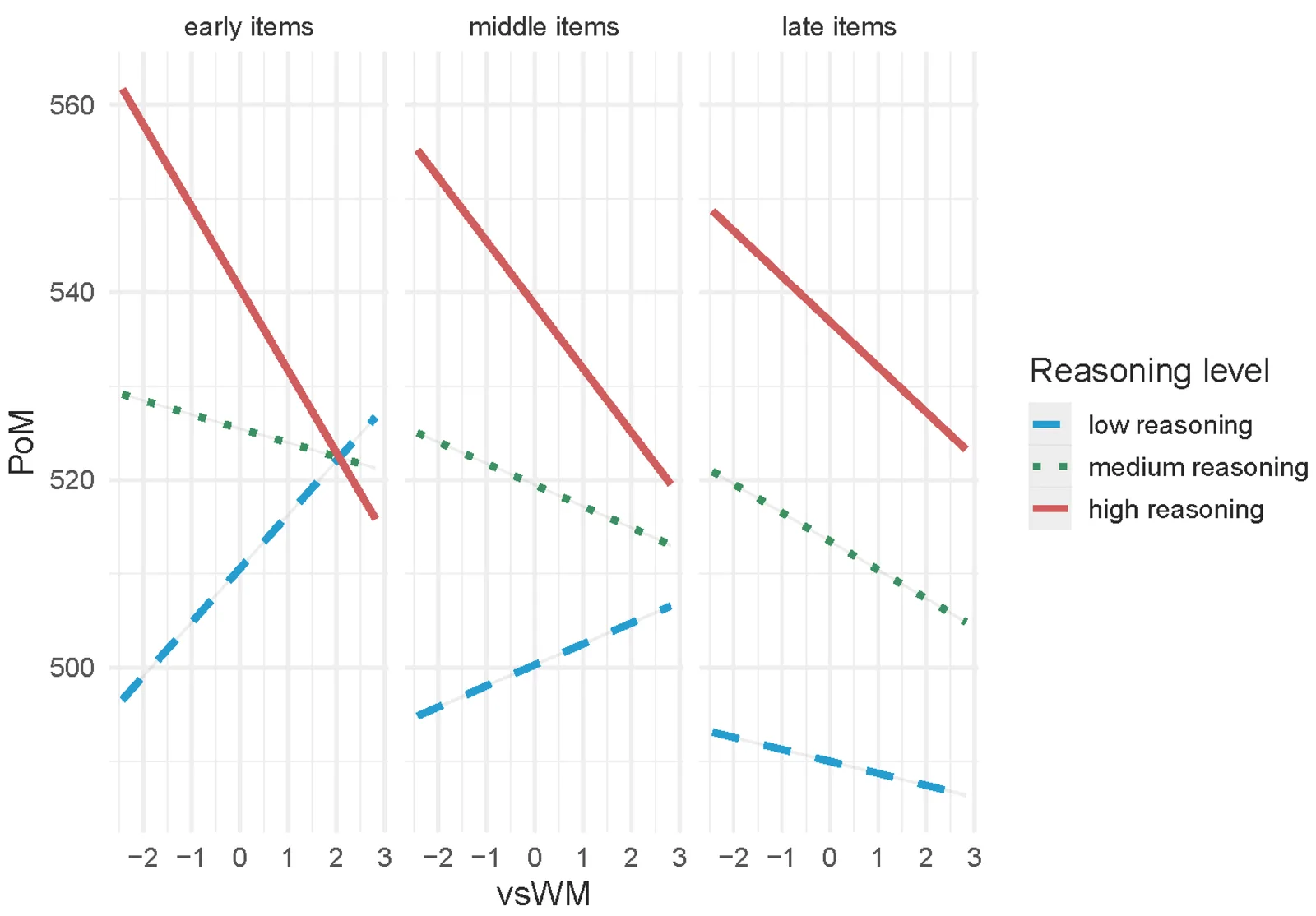
Proportional time on matrix (PoM) across item position, different levels of visuospatial working memory (vsWM) and reasoning ability.

The posterior estimates offered credible evidence for the three-way interaction between vsWM, reasoning ability, and item position. This interaction is illustrated in Figure 7, where instead of item difficulty, vsWM is displayed on the x-axis. For later items (third panel), PoM decreased with higher vsWM regardless of participants’ reasoning ability. Yet for items presented in the middle or at the beginning of the test, the relation between PoM and vsWM changes for participants with low reasoning ability.

For this model with PoM as the outcome variable, a weakly informative prior was specified for the random-effects correlation matrix. This prior mildly favored correlations near zero, while allowing substantial correlations when supported by the data. This was done to improve sampling efficiency, as the uniform prior resulted in low effective sample sizes and slightly inflated $\hat{R}$ values.

The random effect of item position for PoM was large compared to the effect of item difficulty, indicating considerably larger heterogeneity across participant slopes regarding item position (see Figure 5).

Further, random effects revealed a credible, albeit small, positive correlation between the intercept of PoM and item position, indicating that participants spending, on average, less time investigating the matrix experienced a more pronounced (even sharper drop, steeper slope) effect of item position. Vice versa, participants spending more time investigating the matrix showed a dampened effect of item position. Hence, average performance was related to individual changes as the test progressed (item position). Average performance in this context meant items positioned in the middle of the test, with average difficulty and participants exhibiting mean levels of reasoning ability and vsWM.

### 3.3. Proportional Time to First Fixation on Response Alternatives

**Figure 8:**
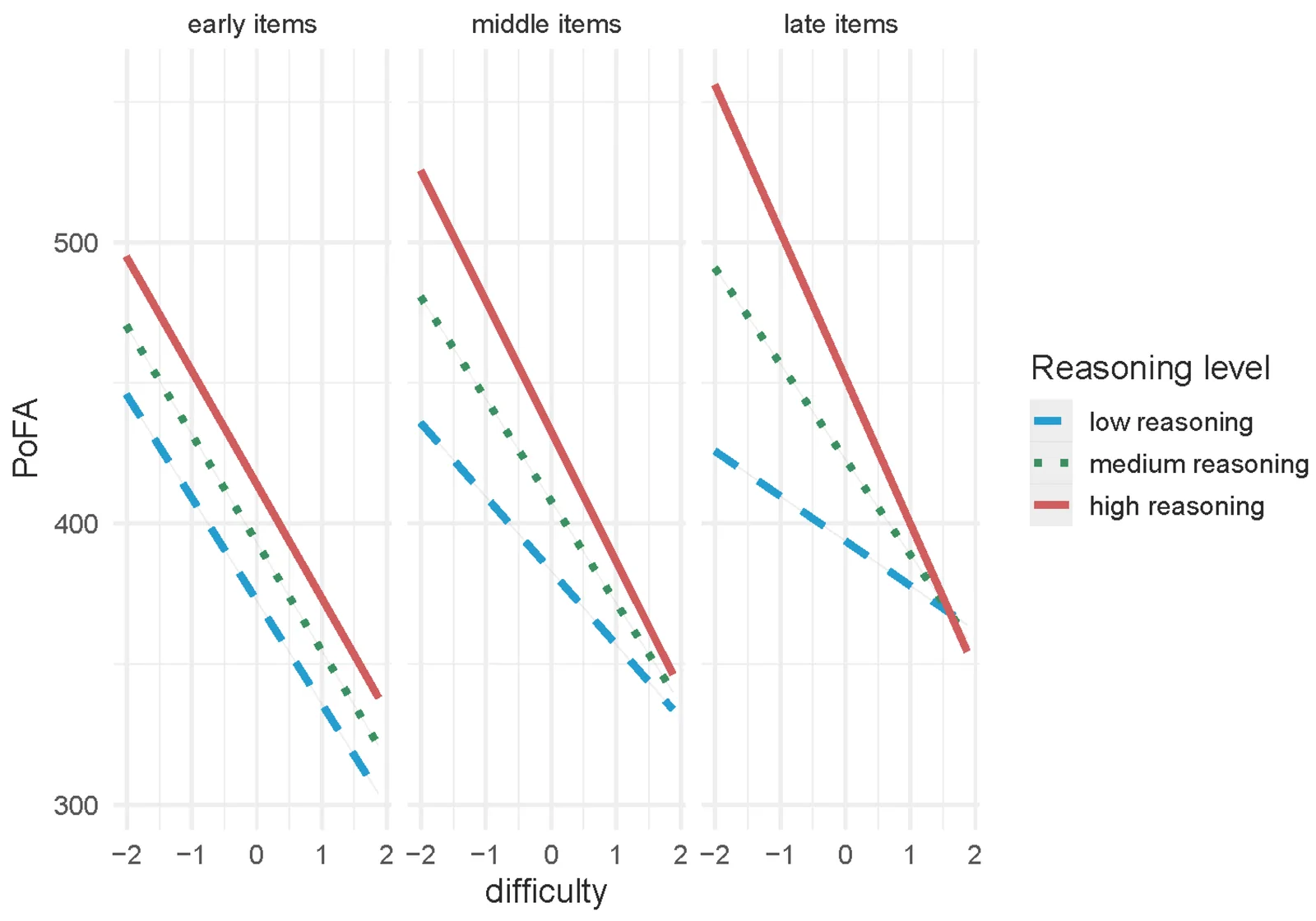
Proportional time to first fixation on response alternatives (PoFA) displayed across item position, varying item difficulty and different levels of reasoning ability.

For PoFA, reasoning ability and item position exhibited a credible positive effect, while no such effect emerged for item difficulty and vsWM (see Table 5). As displayed in Figure 8, participants fixated early on the response alternatives when items were more difficult. For early items, this change in PoFA was similar for all participants, with participants exhibiting higher reasoning ability taking somewhat longer before inspecting response alternatives for the first time. When items were presented in the middle of the test or late in the test, this pattern changed. This pattern change in problem-solving behavior was supported by the credible three-way interaction of reasoning, item difficulty, and item position. For participants with high reasoning ability, the difference in PoFA between difficult and easy items seemed to increase as the test progressed. The opposite occurred for participants with low reasoning ability, where the difference shrunk.

**Figure 9:**
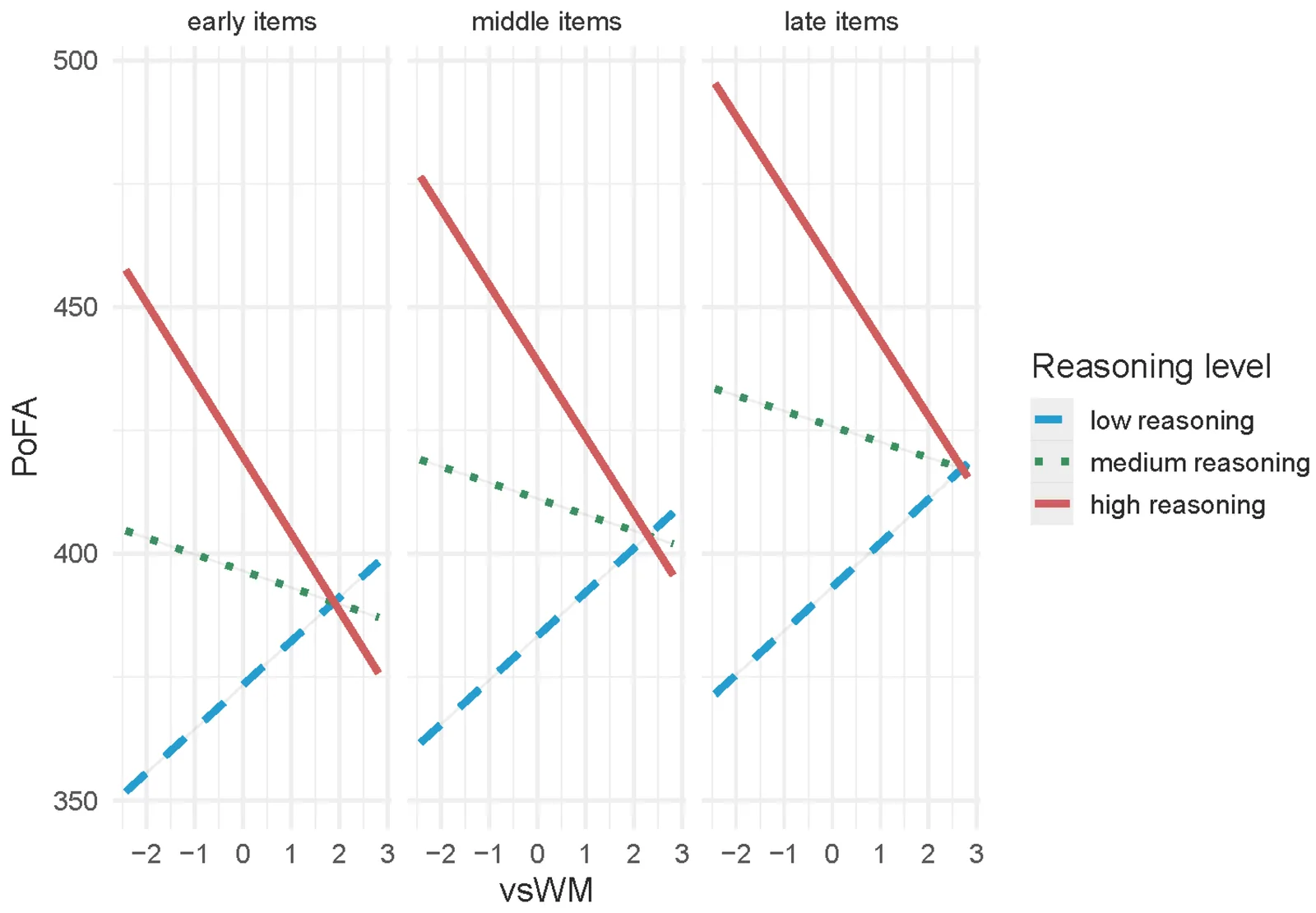
Proportional time to first fixation on response alternatives (PoFA) across item position, different levels of visuospatial working memory (vsWM), and reasoning ability.

Further, the credible four-way interaction indicates that the three-way interaction between reasoning ability, item difficulty, and item position varies systematically as a function of vsWM. Specifically, for participants with higher vsWM, differences in PoFA across item position were more strongly modulated by reasoning ability than for participants with lower vsWM, as illustrated in Figure 9. Importantly, this pattern reflects a conditional effect that emerges only when the interaction among all four predictors is taken into account. Accordingly, the slopes shown in Figure 9 represent a specific slice of the model and additionally vary as a function of item difficulty.

As depicted in Figure 5, the posterior interval estimates of the random effects indicate a credible amount of variability between participants regarding their average PoFA and their slopes regarding item difficulty and item position. Further, a credible positive correlation between item position and the intercept emerged. This indicates that participants with, on average, higher PoFA are also more likely to show variation in their problem-solving behavior regarding item position.

## 4. Discussion

The aim of this study was to explore problem-solving behavior, measured by three different eye-tracking metrics, and how it is influenced by individual differences in reasoning ability and vsWM. Further, the study aimed to investigate how test characteristics such as item difficulty and item position can account for additional variance in problem-solving behavior.

For all three eye-tracking metrics, the models revealed credible main effects for reasoning ability. With higher reasoning ability (and mean vsWM), participants exhibited lower ToR. They spent proportionally more time investigating the problem matrix and took longer before looking at response alternatives for the first time for items with mean item difficulty and presented in the middle of the test. Hence, current results support the finding that with higher reasoning ability, participants are more likely to engage in CM (e.g., Gonthier et al., 2024; Laurence et al., 2023; Vigneau et al., 2006).

Further, results highlight that higher reasoning ability also coincides with larger changes in problem-solving behavior, as previously reported (e.g., Diu et al., 2025; Wang & Zhan, 2025). This pattern is made evident by several credible interactions. For example, with increasing reasoning ability, participants spend increasingly more time from item to item investigating the problem matrix. This indicates an even larger adaptation of problem-solving behavior during test taking. This also occurs with increasing item difficulty. Participants with higher reasoning ability scores spend even more time (steeper slope) investigating the problem matrix when difficulty increased. Regarding ToR, the interaction between reasoning and item difficulty was also credible, indicating that participants toggle less when item difficulty increases. These interactions indicate more CM behavior with increasing item difficulty for individuals with higher reasoning scores.

However, for PoFA, the credible interaction indicates that with increasing item difficulty, the effect of reasoning decreases. This means that participants with high reasoning ability seem less affected by item difficulty regarding their PoFA. Hence, while ToR and PoM are in line with previous research (e.g., Liu et al., 2023) indicating that individuals with higher reasoning ability adapt their behavior to a stronger degree in response to test characteristics, effects on PoFA show the opposite.

Regarding vsWM, current results are not straightforward either. No main effect for vsWM occurred, but a credible interaction with vsWM and reasoning ability was observed in the posterior estimates of ToR. This shows that vsWM has a moderating role when predicting ToR with reasoning ability. With high reasoning ability, ToR decreased, yet this decrease became notably smaller when vsWM was high. This is somewhat contradictory to the assumption that with higher WMC, an individual is better able to apply CM (e.g., Gonthier & Thomassin, 2015; Wang & Zhan, 2025). Present results suggest that the effect of reasoning ability coinciding with more CM is diminished when vsWM is high. Hence, in interaction with reasoning ability, vsWM reduces the use of CM, although this is only evident for ToR and not the other two eye-tracking metrics.

Regarding PoM, the only credible effect for vsWM emerged in the three-way interaction with reasoning ability and item position. While the main effect of item position indicated that participant spend less time on the problem matrix as the test progressed, reasoning ability itself and the interaction between reasoning ability and item position provided credible evidence that participants spend more time on the matrix as the test progressed. The credible three-way interaction with vsWM indicated that this effect was even larger (steeper slope) for individuals with high vsWM.

These results could indicate that when participants are able to hold, maintain, and manipulate objects in their mind very well, they make use of this skill as the test progresses by investigating the problem matrix for longer. However, the extent to which they engage in this behavior is moderated by the individual’s reasoning ability. Or vice versa, with higher reasoning ability, participants spent more time investigating the matrix as the test progresses, yet the extent to which they engage in this problem-solving behavior depends on vsWM ability. While this effect seems similar across item difficulty, item difficulty itself also impacts PoM.

More difficult items coincide with longer matrix inspection times, and this increase in time was even larger for items presented later in the test, as indicated by the credible interaction between item difficulty and item position. Hence, the combination of item difficulty and item position coincides with more CM, as participants spent more time investigating the matrix. The interaction of difficulty and position was also credible in predicting ToR, where posterior estimates also indicate problem-solving behavior more in line with CM, meaning fewer toggles.

The complexity of problem-solving behavior is highlighted by the four-way interaction predicting PoFA. Not only did the interaction of item difficulty and item position depend on reasoning ability, but it was further moderated by vsWM. This means that with increasing reasoning ability, PoFA increases, yet this effect shrinks with increasing item difficulty and again credibly shrinks when items are presented later in the test. Further, this effect is more evident with increasing vsWM. Hence, the positive effects of reasoning ability and item position are dampened as they interact with each other, and additionally with item difficulty and vsWM.

The random effects analyses revealed substantial interindividual variability in problem-solving behavior, particularly with respect to item position (see Figure 5). Compared to item difficulty, item position showed greater heterogeneity in participant slopes across all three eye-tracking metrics. This indicates that participants differed more strongly in how their behavior evolved over the course of the test than in their responses to increasing item difficulty.

For PoM, the positive correlation between the intercept and the item position slope suggests that participants who spend more time investigating the matrix on an average item also show a stronger effect of item position. This means an even steeper decrease of the time spent investigating the matrix as the test progresses.

Regarding PoFA, posterior intervals for random effects revealed a positive correlation between intercept and item position. This suggests that participants who take more time until they fixate on the response alternatives for the first time, experience a stronger effect of item position. In this case, it takes them even longer before they look at response alternatives as the test progresses.

The correlations of random item position slopes and the intercept may be consistent with the meaning-making and learning hypotheses (Knowles et al., 1996; Ren et al., 2014; Schweizer et al., 2020; von Gugelberg et al., 2025). As the test progresses, some individuals may increasingly recognize that successful item solution depends on identifying the underlying rules of the problem matrix. Consequently, they spend more time inspecting the matrix before turning to the response alternatives (PoFA) and devote more overall attention to the matrix (PoM). This increased focus on rule identification may benefit both current and subsequent items if participants learn that similar rules recur throughout the test. Future studies could test this interpretation more directly by incorporating learning tasks while simultaneously examining individual differences in problem-solving behavior.

It is important to highlight that while all three eye-tracking metrics are indices for problem-solving behavior, ToR and PoM reflect a summary of specific behavior occurring while solving an item from start to finish. PoFA, on the other hand, captures only the problem-solving behavior occurring at the very beginning of solving an item. Hence, ToR and PoM also capture strategic behavior individuals engage in just moments before submitting an answer. For example, they may engage in double checking. Double checking would increase ToR, but also result in more PoM, while not affecting PoFA.

Regarding fixed effects, only PoM and ToR showed an item position × difficulty interaction, while all three problem-solving behavior metrics showed a reasoning × difficulty interaction. This could indicate that the difficulty × item position interaction might be due to specific eye movements, happening at the end of the problem-solving passage of each item. Both interactions indicate more CM. This means that, with increasing item difficulty, more time was spent on the problem matrix and less toggling occurred. The latter makes double-checking behavior an unlikely candidate for explaining the phenomenon, since double-checking would lead to frequent toggling between the problem matrix and the response alternatives.

The meta-reasoning framework (Ackerman & Thompson, 2017) highlights different stages in the problem-solving behavior of reasoning tasks. Delving into this framework might offer some insight as to how these eye-tracking metrics potentially capture different stages of the problem-solving behavior. According to this framework, individuals make an initial judgment of solvability, gauging whether the problem seems solvable and whether to give up or seek external help. This initial judgment of solvability is then continuously updated by how confident the individual feels about solving the item successfully. Low confidence in success might lead to giving up or opting out of answering (e.g., Ackerman, 2026; Law et al., 2022). As there is no true option of doing so in common reasoning tests, individuals might turn to guessing or other forms of disengagement (e.g., Nagy et al., 2023).

While the meta-reasoning framework predicts behaviors such as disengagement, opting out, or rapid guessing, the present data provide little evidence for such processes. Item latencies decreased slightly across the test, but accuracy did not (Figure 2 and Figure 3), making a systematic increase of disengagement unlikely. Future studies could include an “I don’t know” option to assess these behaviors more directly. Beyond its methodological utility, such a response may also provide insight into how individuals deal with uncertainty during reasoning test performance.

For example, selecting “I don’t know” could reflect a genuine lack of knowledge, a metacognitive evaluation of one’s uncertainty, or a deliberate strategy to avoid committing to an answer. Investigating which of these processes underlies an “I don’t know” response may contribute to a better understanding of the cognitive and metacognitive mechanisms involved in reasoning test performance.

The meta-reasoning framework further suggests that strategy changes are linked to changes in confidence during problem solving. This possibility is particularly relevant as confidence judgments have been shown to influence reasoning performance (Choo & Double, 2026; Double & Birney, 2017). Future studies combining eye tracking and confidence measures may therefore help clarify how confidence contributes to strategy adaptation during test taking.

Potential for such an endeavor has already been demonstrated by Rodrigues et al. (2026). Their study showed how different gaze transitions were related to individual differences in self-rated metacognitive abilities and task performance (i.e., reasoning task and sequential multitasking). Further, such an investigation in light of the meta-reasoning framework could possibly also shed light on changes in problem-solving behavior happening while solving a single item, as found in Wang and Zhan (2025).

Previous studies have further shown that individual differences such as need for cognition, impulsivity, neuroticism, and confidence-related processes can moderate item-position effects and problem-solving behavior (Birney et al., 2017; Gonthier & Roulin, 2020; Lozano, 2015). These findings suggest that future models of problem-solving behavior should incorporate a broader range of cognitive and personality characteristics.

Educational experiences may also influence how individuals approach reasoning problems. Schneider et al. (2020) showed that teaching the underlying rules of a reasoning test improved performance, while Mitchum and Kelley (2010) found that instruction in CM enhanced metacognitive monitoring. These findings suggest that problem-solving behavior may be additionally shaped by learned strategies rather than cognitive abilities alone. Investigating how instructional approaches influence eye-movement patterns and strategy use during reasoning tasks may therefore be a promising avenue for future research.

Hence, to further the understanding of reasoning ability and individual differences therein, it is advisable not only to broaden the scope by including different personality traits and metacognitive processes, but also to implement a statistical approach that can account for an item-position effect such as the bifactor modeling approach (Schweizer, 2006), the latent class growth models as in Wang and Zhan (2025) or variations of multilevel models (see Birney & Beckmann, 2022).

Additionally, utilizing eye-tracking data to shed light on individual differences in problem-solving behavior seems to be a promising approach. Such data hold an abundance of information, providing numerous possibilities for fine-grained analyses, particularly with respect to capturing within-item dynamics and strategy shifts. For example, Wang and Zhan (2025) introduced a method to divide fixation patterns into small snippets of specific eye-movement sequences. This allowed for a detailed account of individual differences in problem-solving behavior within a single item.

### Limitations and Future Directions

As the present study was exploratory in nature, no specific effect was targeted within a clearly defined experimental design. Consequently, the observed patterns should be interpreted with caution, as they were not derived from hypothesis-driven testing. Future studies should aim to address specific effects within more targeted experimental designs and with clearly formulated hypotheses, allowing for more direct conclusions regarding the mechanisms underlying problem-solving behavior.

Further, only a single, relatively simple measure of vsWM was implemented. While this measure exhibited correlations comparable to those reported in previous research, it remains unclear to what extent the present results generalize to more complex measures of working memory capacity. The correlation of vsWM with reasoning ability in the present study was similar to what Raden and Jarosz (2022) reported, where variations of operation span tasks were used, as well as to findings by Laurence et al. (2023), who also implemented the CBT. However, the use of a single measure limits the ability to fully capture the multidimensional nature of working memory and may partly account for deviations across findings.

Therefore, it would be of interest to examine whether the observed patterns would change when more complex measures are included. In particular, complex updating tasks might yield informative results, as the meta-reasoning framework (Ackerman & Thompson, 2017) suggests continuous updating of solvability judgments during problem solving. Individual differences in the efficiency of this updating process may play an important role in decision making and the adaptation of problem-solving strategies.

Further, the generalizability of current results is limited. The present sample was larger and covered a broader age range than previous studies investigating problem-solving behavior in reasoning tests by means of eye-tracking. The sample also included individuals without higher levels of formal education. Nonetheless, the sample remains far from representative of the general population, limiting the generalizability of the results.

## 5. Conclusions

Results confirm reasoning ability as a credible main effect in predicting the use of CM reflected by lower ToR, higher PoM, and higher PoFA. Additionally, a hallmark of problem-solving behavior for high reasoning ability individuals is adaptive behavior regarding test characteristics, including not only item difficulty, as frequently reported, but also item position. The latter showed remarkable between-participant slope heterogeneity. This heterogeneity was not accounted for by reasoning ability or vsWM, suggesting that problem-solving behavior in reasoning tests reflects more than cognitive ability alone. Rather, the present findings indicate that strategy use emerges from a dynamic interplay between individual characteristics and test characteristics, with participants differing markedly in how their behavior changes throughout the test.

As a final remark, the present results largely replicated previously reported correlations between eye-tracking metrics and reasoning ability, although the observed effects were generally smaller. Number of Toggles remained the exception, showing substantial inconsistency across studies (see Table 1). Notably, similar correlations have nevertheless been accompanied by markedly different conclusions regarding changes in problem-solving behavior during test taking (i.e., increases vs. decreases in CM). This suggests that correlations alone may obscure the influence of individual differences and test characteristics, both of which appear crucial for understanding variability in reasoning test performance and problem-solving behavior during test taking.

## Figures and Tables

**Table 1:** Correlations between different eye-tracking metrics and reasoning ability.

	Gonthier et al. (2024) *	Laurence et al. (2018)	Laurence et al. (2023)	Niebaum and Munakata (2023)	Raden and Jarosz (2022)	Rivollier et al. (2021) *	Vigneau et al. (2006)
**Sample Size**	**474 (children & adults)**	**34**	**69** **(study 2)**	**51** **(adults)**	**53/58** **(study 1)**	**130**	**55**
**Number of Items** **Reasoning Test**	**48** **SPM (B** **–** **E)**	**18** **WMT-2**	**18** **WMT-2**	**24** **APM**	**36** **APM**	**12** **APM**	**14** **APM**
**Item Latency**	**0.35**	**0.57**	**0.31**	**/**	**/**	**0.63**	0.03
**Number of Toggles**	**0.17**	0.10	−0.10	0.18	**/**	0.02	**−0.27**
**ToR**	**−0.18**	**−0.68**	**−0.50**	**−0.56**	**−0.47/−0.46**	**−0.55**	**−0.43**
**Time on Matrix**	**/**	**0.55**	**0.34**	**/**	**/**	**0.65**	0.08
**PoM**	**0.73**	0.26	**0.36 ^+^**	**0.48**	**/**	**0.56**	**0.48**
**Time to First Fixation on** **Response Alternatives**	**0.48**	**0.55**	**0.32**	**/**	**/**	**/**	**0.41**
**PoFA**	**0.47**	**0.55**	**/**	**0.49**	**0.35/0.54**	**0.70**	**/**

Note. Given are reported correlations between eye-tracking metrics and reasoning ability of different studies. Bold values are significant. * These studies had a setup to mimic eye-tracking. ^+^ Here the ratio between time on matrix and time on response alternatives was reported. APM = Raven’s Advanced Progressive Matrices; SPM = Raven’s Standard Progressive Matrices; WMT = Wiener Matrizen Test.

**Table 2:** Correlation matrix between measured variables.

	Reasoning Ability	Item Latency	Number of Toggles	ToR	Time on Matrix	PoM	Time to First Fixation on Response Alternatives	PoFA
Item Latency	0.15							
Number of Toggles	−0.28	0.47						
ToR	−0.44	−0.48	0.45					
Time on Matrix	0.16	0.87	0.40	−0.46				
PoM	0.23	0.13	*−0.07*	−0.25	0.54			
Time to First Fixation on Response Alternatives	0.26	0.53	−0.25	−0.71	0.54	0.26		
PoFA	0.23	−0.21	−0.73	−0.51	−0.10	0.26	0.65	
vsWM	0.41	*0.06*	−0.16	−0.24	*0.08*	*0.08*	*0.09*	*0.08*

Note. Correlations between item latency, reasoning ability, eye-tracking metrics and visuospatial working memory (vsWM). Italic values are not significant. ToR = toggle rate; PoM = proportional time on problem matrix; PoFA = proportional time to first fixation on response alternatives.

**Table 3:** Posterior estimates of fixed effects predicting toggle rate from Bayesian analysis.

Predictor	Estimate	SE	CI Lower	CI Upper
**Intercept**	**3.03**	**0.02**	**3.00**	**3.06**
vsWM	−0.01	0.02	−0.04	0.03
**reasoning**	**−0.11**	**0.02**	**−0.15**	**−0.08**
**difficulty**	**−0.10**	**0.01**	**−0.12**	**−0.09**
item position	0.01	0.01	−0.01	0.02
**vsWM: reasoning**	**0.06**	**0.01**	**0.03**	**0.09**
vsWM: difficulty	−0.01	0.01	−0.02	0.00
**reasoning: difficulty**	**−0.03**	**0.01**	**−0.04**	**−0.02**
vsWM: item position	−0.00	0.01	−0.02	0.01
reasoning: item position	0.00	0.01	−0.01	0.02
**difficulty: item position**	**−0.02**	**0.01**	**−0.03**	**−0.01**
vsWM: reasoning: difficulty	0.00	0.00	−0.01	0.01
vsWM: reasoning: item position	−0.00	0.01	−0.01	0.01
vsWM: difficulty: item position	−0.00	0.01	−0.02	0.01
reasoning: difficulty: item position	0.00	0.01	−0.01	0.01
vsWM: reasoning: difficulty: item position	−0.00	0.00	−0.01	0.01

Note. Toggle Rate was multiplied by 100 prior to running the model to ease interpretation. Credible intervals (CI) highlighted in bold do not include zero and reflect effects with strong posterior support. All parameters demonstrated satisfactory convergence ($\hat{R}$ < 1.01).

**Table 4:** Posterior estimates of fixed effects predicting proportional time on problem matrix from Bayesian analysis.

Predictor	Estimate	SE	CI Lower	CI Upper
**Intercept**	**520.31**	**4.94**	**510.86**	**530.12**
vsWM	−2.31	5.15	−12.50	7.64
**reasoning**	**19.21**	**5.15**	**9.41**	**29.44**
**difficulty**	**9.78**	**0.86**	**8.09**	**11.46**
**item position**	**−6.45**	**1.43**	**−9.29**	**−3.68**
vsWM: reasoning	−4.63	4.43	−13.32	4.06
vsWM: difficulty	−0.38	0.89	−2.13	1.36
**reasoning: difficulty**	**4.72**	**0.90**	**2.98**	**6.46**
vsWM: item position	−0.85	1.47	−3.73	2.05
**reasoning: item position**	**4.96**	**1.48**	**2.06**	**7.89**
**difficulty: item position**	**7.63**	**0.91**	**5.85**	**9.42**
vsWM: reasoning: difficulty	−0.07	0.76	−1.59	1.41
**vsWM: reasoning: item position**	**3.17**	**1.26**	**0.70**	**5.60**
vsWM: difficulty: item position	0.87	0.94	−0.97	2.72
reasoning: difficulty: item position	0.61	0.94	−1.25	2.43
vsWM: reasoning: difficulty: item position	−0.10	0.81	−1.69	1.50

Note. Credible intervals (CI) highlighted in bold do not include zero and reflect effects with strong posterior support. All parameters demonstrated satisfactory convergence ($\hat{R}\;$ < 1.01).

**Table 5:** Posterior estimates of fixed effects predicting proportional time to first fixation on response alternatives from Bayesian analysis.

Predictor	Estimate	SE	CI Lower	CI Upper
**Intercept**	**408.52**	**7.25**	**394.30**	**422.80**
vsWM	−3.50	7.56	−18.36	11.17
**reasoning**	**27.22**	**7.61**	**12.37**	**42.14**
**difficulty**	**−36.06**	**2.84**	**−41.60**	**−30.56**
**item position**	**17.10**	**3.43**	**10.35**	**23.79**
vsWM: reasoning	−12.11	6.62	−25.07	0.95
vsWM: difficulty	−0.74	2.92	−6.42	5.01
**reasoning: difficulty**	**−10.08**	**2.93**	**−15.87**	**−4.36**
vsWM: item position	0.31	3.49	−6.61	7.19
reasoning: item position	4.91	3.54	−2.03	11.94
difficulty: item position	1.99	2.96	−3.76	7.74
vsWM: reasoning: difficulty	0.61	2.54	−4.37	5.62
vsWM: reasoning: item position	−0.31	3.06	−6.32	5.71
vsWM: difficulty: item position	3.04	3.04	−2.96	8.97
**reasoning: difficulty: item position**	**−8.08**	**3.05**	**−13.99**	**−2.13**
**vsWM: reasoning: difficulty: item position**	**−6.02**	**2.66**	**−11.32**	**−0.81**

Note. Credible intervals (CI) highlighted in bold do not include zero and reflect effects with strong posterior support. All parameters demonstrated satisfactory convergence ($\hat{R}$ < 1.01).

## Data Availability

Data is available on OSF at: https://osf.io/sxjb2/overview?view_only=08f3353431194817bc3366ad4bfcb468 (accessed on 20 August 2026).

## Notes

1 Whenever the calibration did not work after three attempts, the study was continued without eye-tracking to ensure the same treatment of all participants and not cause unnecessary discomfort.

2 Some participants showed fixations clustered at the top of the screen, or only very sparse fixations on screen, or were scattered over the whole screen with no fixations on response alternatives.

3 For all models, higher item difficulty values correspond to higher difficulty, meaning less participants were able to answer the item correctly. Only for Figure 2, item difficulty was calculated as the difficulty index *P_i_* for ease of comparisons with other studies.

## Supplementary Materials

Supporting information can be downloaded from OSF at: https://osf.io/sxjb2/overview?view_only=08f3353431194817bc3366ad4bfcb468 (accessed on 20 August 2026).

## Abbreviations

The following abbreviations are used in this manuscript:

CM	constructive matching
RE	response elimination
ToR	toggle rate
PoM	proportional time on matrix
PoFA	proportional time to first fixation on response alternatives
WMC	working memory capacity
vsWM	visuospatial working memory
FM	figural matrices
CBT	Corsi block-tapping task
AOI	areas of interest

## References

[B1-jintelligence-14-00206] Ackerman R. (2026). It’s time to opt out: Metacognitive analysis of time regulation under uncertainty.

[B2-jintelligence-14-00206] Ackerman R., Thompson V. A. (2017). Meta-reasoning: Monitoring and control of thinking and reasoning. Trends in Cognitive Sciences.

[B3-jintelligence-14-00206] Bethell-Fox C. E., Lohman D. F., Snow R. E. (1984). Adaptive reasoning: Componential and eye movement analysis of geometric analogy performance. Intelligence.

[B4-jintelligence-14-00206] Birney D. P., Beckmann J. F. (2022). Intelligence is cognitive flexibility: Why multilevel models of within-individual processes are needed to realise this. Journal of Intelligence.

[B5-jintelligence-14-00206] Birney D. P., Beckmann J. F., Beckmann N., Double K. S. (2017). Beyond the intellect: Complexity and learning trajectories in Raven’s progressive matrices depend on self-regulatory processes and conative dispositions. Intelligence.

[B6-jintelligence-14-00206] Bojko A. (2013). Eye tracking the user experience: A practical guide to research.

[B7-jintelligence-14-00206] Bürkner P.-C. (2017). brms: An R package for Bayesian multilevel models using Stan. Journal of Statistical Software.

[B8-jintelligence-14-00206] Carpenter P. A., Just M. A., Shell P. (1990). What one intelligence test measures: A theoretical account of the processing in the Raven progressive matrices test. Psychological Review.

[B9-jintelligence-14-00206] Cheyette S. J., Piantadosi S. T. (2024). Response to difficulty drives variation in IQ test performance. Open Mind.

[B10-jintelligence-14-00206] Choo R., Double K. S. (2026). Metacognitive sensitivity moderates reactivity to confidence ratings. Thinking & Reasoning.

[B11-jintelligence-14-00206] Deary I. J., Strand S., Smith P., Fernandes C. (2007). Intelligence and educational achievement. Intelligence.

[B12-jintelligence-14-00206] Diu A., Batty M., Bouvet L. (2025). Visual strategies in solving Raven’s matrices: Insights from autism. Research in Autism.

[B13-jintelligence-14-00206] Double K. S., Birney D. P. (2017). Are you sure about that? Eliciting confidence ratings may influence performance on Raven’s progressive matrices. Thinking & Reasoning.

[B14-jintelligence-14-00206] Gonthier C., Harma K., Gavornikova-Baligand Z. (2024). Development of reasoning performance in Raven’s matrices is grounded in the development of effective strategy use. Journal of Experimental Psychology: General.

[B15-jintelligence-14-00206] Gonthier C., Roulin J.-L. (2020). Intraindividual strategy shifts in Raven’s matrices, and their dependence on working memory capacity and need for cognition. Journal of Experimental Psychology: General.

[B16-jintelligence-14-00206] Gonthier C., Thomassin N. (2015). Strategy use fully mediates the relationship between working memory capacity and performance on Raven’s matrices. Journal of Experimental Psychology: General.

[B17-jintelligence-14-00206] Jarosz A. F., Raden M. J., Wiley J. (2019). Working memory capacity and strategy use on the RAPM. Intelligence.

[B18-jintelligence-14-00206] Jastrzębski J., Ciechanowska I., Chuderski A. (2018). The strong link between fluid intelligence and working memory cannot be explained away by strategy use. Intelligence.

[B19-jintelligence-14-00206] Kane M. J., Hambrick D. Z., Conway A. R. A. (2005). Working memory capacity and fluid intelligence are strongly related constructs: Comment on Ackerman, Beier, and Boyle (2005). Psychological Bulletin.

[B20-jintelligence-14-00206] Knowles E. S., Coker M. C., Scott R. A., Cook D. A., Neville J. W. (1996). Measurement-induced improvement in anxiety: Mean shifts with repeated assessment. Journal of Personality and Social Psychology.

[B21-jintelligence-14-00206] Kyllonen P., Hartman R., Sprenger A., Weeks J., Bertling M., McGrew K., Kriz S., Bertling J., Fife J., Stankov L. (2019). General fluid/inductive reasoning battery for a high-ability population. Behavior Research Methods.

[B22-jintelligence-14-00206] Laurence P. G., Jana T. A., Bunge S. A., Macedo E. C. (2023). Eye gaze patterns during reasoning provide insights regarding individual differences in underlying cognitive abilities. Journal of Intelligence.

[B23-jintelligence-14-00206] Laurence P. G., Mecca T. P., Serpa A., Martin R., Macedo E. C. (2018). Eye movements and cognitive strategy in a fluid intelligence test: Item type analysis. Frontiers in Psychology.

[B24-jintelligence-14-00206] Law M. K., Stankov L., Kleitman S. (2022). I choose to opt-out of answering: Individual differences in giving up behaviour on cognitive tests. Journal of Intelligence.

[B25-jintelligence-14-00206] Li C., Ren X., Schweizer K., Wang T. (2022). Strategy use moderates the relation between working memory capacity and fluid intelligence: A combined approach. Intelligence.

[B26-jintelligence-14-00206] Liu Y., Zhan P., Fu Y., Chen Q., Man K., Luo Y. (2023). Using a multi-strategy eye-tracking psychometric model to measure intelligence and identify cognitive strategy in Raven’s advanced progressive matrices. Intelligence.

[B27-jintelligence-14-00206] Lozano J. H. (2015). Are impulsivity and intelligence truly related constructs? Evidence based on the fixed-links model. Personality and Individual Differences.

[B28-jintelligence-14-00206] Martarelli C. S., Mast F. W. (2013). Eye movements during long-term pictorial recall. Psychological Research.

[B29-jintelligence-14-00206] Mitchum A. L., Kelley C. M. (2010). Solve the problem first: Constructive solution strategies can influence the accuracy of retrospective confidence judgments. Journal of Experimental Psychology: Learning, Memory, and Cognition.

[B30-jintelligence-14-00206] Nagy G., Ulitzsch E., Lindner M. A. (2023). The role of rapid guessing and test-taking persistence in modelling test-taking engagement. Journal of Computer Assisted Learning.

[B31-jintelligence-14-00206] Niebaum J., Munakata Y. (2023). The development of relational reasoning: An eyetracking analysis of strategy use and adaptation in children and adults performing matrix completion. Open Mind.

[B32-jintelligence-14-00206] Oberauer K. (2020). Towards a theory of working memory. Working memory: The state of the science.

[B33-jintelligence-14-00206] Peirce J., Gray J. R., Simpson S., MacAskill M., Höchenberger R., Sogo H., Kastman E., Lindeløv J. K. (2019). PsychoPy2: Experiments in behavior made easy. Behavior Research Methods.

[B34-jintelligence-14-00206] Perret P., Dauvier B. (2018). Children’s allocation of study time during the solution of Raven’s progressive matrices. Journal of Intelligence.

[B35-jintelligence-14-00206] Raden M. J., Jarosz A. F. (2022). Strategy transfer on fluid reasoning tasks. Intelligence.

[B36-jintelligence-14-00206] Raven J. C., Raven J., McCallum R. S. (2003). Raven Progressive Matrices. Handbook of nonverbal assessment.

[B37-jintelligence-14-00206] R Core Team (2020). R: A Language and Environment for Statistical Computing.

[B38-jintelligence-14-00206] Ren X., Wang T., Altmeyer M., Schweizer K. (2014). A learning-based account of fluid intelligence from the perspective of the position effect. Learning and Individual Differences.

[B39-jintelligence-14-00206] Rivollier G., Quinton J.-C., Gonthier C., Smeding A. (2021). Looking with the (computer) mouse: How to unveil problem-solving strategies in matrix reasoning without eye-tracking. Behavior Research Methods.

[B40-jintelligence-14-00206] Rodrigues M. d. M., Hodgson T., Laurence P. G., Macedo E. C. (2026). Eye-gaze strategies reveal cognitive variability in a real-world executive function task. Neuropsychology.

[B41-jintelligence-14-00206] Roth B., Becker N., Romeyke S., Schäfer S., Domnick F., Spinath F. M. (2015). Intelligence and school grades: A meta-analysis. Intelligence.

[B42-jintelligence-14-00206] Sackett P. R., Demeke S., Bazian I. M., Griebie A. M., Priest R., Kuncel N. R. (2024). A contemporary look at the relationship between general cognitive ability and job performance. Journal of Applied Psychology.

[B43-jintelligence-14-00206] Schneider B., Becker N., Krieger F., Spinath F. M., Sparfeldt J. R. (2020). Teaching the underlying rules of figural matrices in a short video increases test scores. Intelligence.

[B44-jintelligence-14-00206] Schweizer K. (2006). The fixed-links model for investigating the effects of general and specific processes on intelligence. Methodology.

[B45-jintelligence-14-00206] Schweizer K., Zeller F., Reiß S. (2020). Higher-order processing and change-to-automaticity as explanations of the item-position effect in reasoning tests. Acta Psychologica.

[B46-jintelligence-14-00206] Snow R. E. (1978). Theory and method for research on aptitude processes. Intelligence.

[B47-jintelligence-14-00206] Snow R. E., Snow R. E., Frederico P.-A., Montague W. E. (1980). Aptitude processes. Aptitude, learning, and instruction.

[B48-jintelligence-14-00206] SR Research (2016). Eyelink 1000 plus *[Apparatus and software]*.

[B49-jintelligence-14-00206] Vigneau F., Caissie A. F., Bors D. A. (2006). Eye-movement analysis demonstrates strategic influences on intelligence. Intelligence.

[B50-jintelligence-14-00206] von Gugelberg H. M., Schweizer K., Troche S. J. (2025). Experimental evidence for rule learning as the underlying source of the item-position effect in reasoning ability measures. Learning and Individual Differences.

[B51-jintelligence-14-00206] von Gugelberg H. M., Troche S. J. (2025). Individual differences in strategy and the item-position effect in reasoning ability measures. Journal of Intelligence.

[B52-jintelligence-14-00206] Wang Z., Zhan P. (2025). Eye-tracking-based hidden Markov modeling for revealing within-item cognitive strategy switching. Behavior Research Methods.

[B53-jintelligence-14-00206] Wickham H., Averick M., Bryan J., Chang W., McGowan L., François R., Grolemund G., Hayes A., Henry L., Hester J., Kuhn M., Pedersen T., Miller E., Bache S., Müller K., Ooms J., Robinson D., Seidel D., Spinu V., Yutani H. (2019). Welcome to the tidyverse. Journal of Open Source Software.

[B54-jintelligence-14-00206] Zeller F., Reiß S., Schweizer K. (2017). Is the item-position effect in achievement measures induced by increasing item difficulty?. Structural Equation Modeling: A Multidisciplinary Journal.

